# GDF15 ameliorates sepsis-induced lung injury via AMPK-mediated inhibition of glycolysis in alveolar macrophage

**DOI:** 10.1186/s12931-024-02824-z

**Published:** 2024-05-09

**Authors:** Shasha Lu, Ranran Li, Yunxin Deng, Ju Bai, Bangqi Ji, Yufeng Chu, Yan Xu, Hongping Qu, Xiaosun Guo, Pibao Li, Mei Meng

**Affiliations:** 1 Department of Critical Care Medicine, Ruijin Hospital, Shanghai Jiao Tong University School of Medicine, No. 197, Ruijin Road (No.2), Huangpu District, Shanghai, 200025 P.R. China; 2 The first rehabilitation hospital of Shandong, Linyi, 276000 Shandong P.R. China; 3 Ocean University of China, Qingdao, 266000 Shandong P.R. China; 4 Yantai Affiliated Hospital of Binzhou Medical University, Binzhou, 256600 Shandong P.R. China; 5 Shandong Rehabilitation Hospital, Jinan, 250109 Shandong P.R. China; 6 Department of Critical Care Medicine, The Second Hospital, Cheeloo College of Medicine, Shandong University, Jinan, 250000 P.R. China; 7 Department of Physiology and Pathophysiology, School of Basic Medical Sciences, Cheeloo College of Medicine, Shandong University, Jinan, 250000 P.R. China

**Keywords:** Growth differentiation factor 15(GDF15), Glycolysis, Inflammation, sepsis, Alveolar macrophage

## Abstract

Growth differentiation factor 15 (GDF15) as a stress response cytokine is involved in the development and progression of several diseases associated with metabolic disorders. However, the regulatory role and the underlying mechanisms of GDF15 in sepsis remain poorly defined. Our study analyzed the levels of GDF15 and its correlations with the clinical prognosis of patients with sepsis. In vivo and in vitro models of sepsis were applied to elucidate the role and mechanisms of GDF15 in sepsis-associated lung injury. We observed strong correlations of plasma GDF15 levels with the levels of C-reactive protein (CRP), procalcitonin (PCT), lactate dehydrogenase (LDH), and lactate as well as Sequential Organ Failure Assessment (SOFA) scores in patients with sepsis. In the mouse model of lipopolysaccharide-induced sepsis, recombinant GDF15 inhibited the proinflammatory responses and alleviated lung tissue injury. In addition, GDF15 decreased the levels of cytokines produced by alveolar macrophages (AMs). The anti-inflammatory effect of glycolysis inhibitor 2-DG on AMs during sepsis was mediated by GDF15 via inducing the phosphorylation of the α-subunit of eukaryotic initiation factor 2 (eIF2α) and the expression of activating transcription factor 4 (ATF4). Furthermore, we explored the mechanism underlying the beneficial effects of GDF15 and found that GDF15 inhibited glycolysis and mitogen-activated protein kinases (MAPK)/nuclear factor-κB (NF-κB) signaling via promoting AMPK phosphorylation. This study demonstrated that GDF15 inhibited glycolysis and NF-κB/MAPKs signaling via activating AMP-activated protein kinase (AMPK), thereby alleviating the inflammatory responses of AMs and sepsis-associated lung injury. Our findings provided new insights into novel therapeutic strategies for treating sepsis.

**Supplementary Information:**

The online version contains supplementary material available at 10.1186/s12931-024-02824-z.

## Introduction

Sepsis is a life-threatening organ dysfunction caused by the host’s maladjusted response to infection [1]. Lung injury is the most common complication of sepsis, which leads to hypoxia caused by the disorder of gas and blood exchange, aggravating the injury of other organs. Despite advances in ventilation strategies, mortality for sepsis-induced lung injury remains high [2]. The dysregulated immune response are the hallmarks of sepsis-induced lung injury. Alveolar macrophages (AMs) as the main innate immune cells are responsible for the initiation and resolution of the lung immune responses [3]. Upon the onset of sepsis, AMs are activated and secrete a large number of inflammatory factors, such as tumor necrosis factor α (TNFα), interleukin-1β (IL-1β), and interleukin-6 (IL-6), triggering the inflammatory cytokine storm and tissue damage. Therefore, interfering the overwhelming inflammatory response of AMs is considered as an important therapeutic strategy for treating sepsis-related lung injury.

Growth differentiation factor 15 (GDF15), also named as macrophage inhibitory cytokine-1 (MIC1), is a divergent member of the transforming growth factor β (TGFβ) superfamily [4]. As a circulating cytokine, GDF15 has been implicated in multiple biological processes associated with energy homeostasis [5]. GDF15 is a stress-response factor that is involved in several lung diseases including lung cancer, pulmonary fibrosis, and pulmonary arterial hypertension [6–8]. Most recently, GDF15 has been recognized to be an inflammation-related hormone that is essential for surviving infections [9, 10]. By regulating triglyceride metabolism, GDF15 coordinates the host tolerance to inflammatory stimuli [11]. GDF15 deficiency aggravated renal and cardiac injury in sepsis due to increased inflammatory cytokines. According to the above, GDF15 may be involved in the inflammatory response during sepsis-induced lung injury. However, the detailed regulatory effect and underlying mechanisms of GDF15 on AMs in sepsis-induced lung injury remains unexclusive.

Recent studies regarding immunometabolism have reported that macrophage functions are closely related to aerobic glycolysis [12]. Glycolysis inhibition alleviates sepsis-induced inflammation and organ damage [13, 14]. GDF15 is known to play a critical role in energy metabolism [15, 16]. Metformin, which can ameliorate mTOR/HIF1⍺-mediated aerobic glycolysis [17], has been reported to increase circulating levels of GDF15 [18]. However, the regulatory interaction between glycolysis and GDF15 is unknown yet.

In the present study, we found that although the plasma levels of GDF15 were positively correlated with the severity of patients with sepsis, recombinant GDF15 administration in vivo alleviated sepsis-induced systemic inflammation and lung injury. We further demonstrated that glycolysis inhibition induced GDF15 expression in AMs via activating eIF2⍺-ATF4 signaling. Furthermore, GDF15 alleviated inflammatory response of AMs in sepsis-induced lung injury via AMPK activation-mediated inhibition of glycolysis and MAPKs/NF-κB signaling. Our findings reveal the beneficial role of GDF15 in AM inflammation during sepsis, providing a potential therapeutic strategy for treating sepsis-related lung injury.

## Materials and methods

### Patient study protocol

Patients diagnosed with sepsis in the Department of Critical Care Medicine of Shanghai Ruijin Hospital from July 1st, 2021 to May 31st, 2022 were enrolled. This study was approved by the Ethics Committee of Ruijin hospital (20,200,011). The investigation was based on the institution’s guidelines for human studies and conformed to the ethics guidelines of the declaration of Helsinki. Informed consent was obtained from each participant. The enrollment criteria were as follows: (1) 18–80 years old; (2) adherence to the sepsis 3.0 diagnostic criteria; (3) hospital stay > 24 h. The corresponding exclusion criteria were as follows: (1) discharge or death within 24 h after admission; (2) participation in other clinical research; (3) emergency surgery after admission; and (4) malignant tumor; (5) pregnant patients; (6) lack of necessary clinical data. Finally, a total of 12 healthy volunteers, 29 septic patients were enrolled.

### Reagents

2-DG (#S4701), ISRIB (#S0706), Salubrinal (#S2923), GCN2IB (#S8929), C16(#S9668), GSK2656157 (#S7033), and Compound C (#S7840) were purchased from Selleck (shanghai, China). Lipopolysaccharide (LPS, #L2630, E. coli 0111:B4) was purchased from Sigma-Aldrich (St. Louis, MO, USA). Mouse recombinant GDF15 (#10596-GD-025) was purchased from R&D Systems (Minnesota, USA). Pooled mouse ATF4 siRNA (#1. Sense-5’-CUCCCAGAAAGUUUAAUAATT-3’, anti-sense-5’- UUAUUAAACUUUCUGGGAGTT-3’; #2. Sense-5’-GCUGCUUACAUUACUCUAATT-3’, anti-sense-5’-UUAGAGUAAUGUAAGCAGCTT-3’; #. Sense-5’-GUCUCUUAGAUGACUAUCUTT-3’, anti-sense-5’-AGAUAGUCAUCUAAGAGACTT-3’) and pooled mouse GDF15 siRNA (#1. Sense-5’-CUCGAACUCAGAACCAAGUTT-3’, anti-sense-5’-ACUUGGUUCUGAGUUCGAGTT-3’; #2. Sense-5’-GUGGUUCUUAUGCACAGGATT-3’, anti-sense-5’-UCCUGUGCAUAAGAACCACTT-3’; #3. Sense-5’-CUGCUAAUAAAGGUGAGCUTT-3’, anti-sense-5’-AGCUCACCUUUAUUAGCAGTT-3’) were synthesized by GenePharma (Shanghai, China). Antibodies against phosphorylated eIF2α (3597 S), total eIF2α (5324 S), ATF4 (11,815 S), phosphorylated AMPK (2535 S), and AMPK (2532 S) were purchased from cell signaling technology (MA, USA). Antibodies against Glut1(66,290), HK2 (66,974), PFKFB3 (13,123), and PKM2 (60,268) were obtained from Proteintech (Wuhan, China) Antibody against GDF15 (ab105738) was purchased from Abcam (MA, USA).

### Cell culture

The alveolar macrophage cell line MH-S (#ZQ0921, ScienCell, CA, USA) were cultured in RPMI 1640 complete medium supplemented with 10% FBS (AU0600), 1% penicillin/streptomycin, 0.05mM β-mercaptoethanol (#ZQ-206, ScienCell) at 37 °C with 95% humidity and 5% CO_2_. MH-S were pretreated with the indicated inhibitors followed by stimulation with 1 µg/mL LPS for the indicated time periods to imitate inflammation.

### siRNA transfection

MH-S were seeded at a density of 50–70%. HiPerFect (301,704, Qiagen, Germany) was used as the transfection reagent according to the manufacturer’s instructions. Cells were treated with different stimuli 48 h after transfection and were harvested for further analysis.

### RNA sequencing

Total RNA was extracted from MH-S stimulated with LPS in the absence and presence of 2-DG. The concentration and purity of isolated RNA were measured using a ND-800 spectrophotometer (Thermo Fisher Scientific, DE, USA). The RNA libraries were constructed using a Truseq RNA Library Prep Kit. Sequencing was carried out using a 2 × 150 bp PE configuration. The clean data (reads) were mapped using Hisat2 (version 2.1.0). Then, we performed gene expression analysis using RSEM (version 1.3.1). Differential expression analysis among different groups was conducted using DESeq2, and then |FC| >2 and FDR < 0.05 were determined as thresholds for differentially expressed genes (DEGs). Gene Ontology (GO) analysis was used to determine the significant biological processes of a particular gene set (*q* < 0.05). The Kyoto Encyclopedia of Genes and Genomes (KEGG) analysis was used to identify the most significant signaling pathways involved in BMS-303,141-regulated genes (adjusted *P* < 0.05). The Venn analysis was used to calculate the number of genes in different gene sets.

### Murine model

Male C57BL/6 mice (6–8 weeks, 20–25 g) were obtained from Charles River (Beijing, China) and randomly divided into experimental groups. The mice were intraperitoneally injected with LPS (5 mg/kg body weight) to induce endotoxemia. In the intervention group, the mice were intraperitoneally injected with mouse recombinant GDF15 (50 ng/kg body weight), salubrinal (1 mg/kg body weight), or 2-DG (500 mg/kg body weight) 1 h before LPS injection. Vehicle control mice were intraperitoneally injected with 100 µL of 0.9% NaCl. 16 h after LPS challenge, the mice were anesthetized and blood samples were taken. Organs were snap frozen or fixed with formalin for further examination. Frozen organs were stored at -80 °C. The protocols for animal experiments were approved by the Animal Ethics Committee of Ruijin Hospital Affiliated to Shanghai Jiao Tong University School of Medicine (No. 092) and were in line with the International Guidelines for Care and Use of Laboratory Animals (National Academy of Sciences Health Publication No. 85–23, revised in 1996). All animal experiments were conducted according to the principles of laboratory animal care.

### Isolation of mouse alveolar macrophages from BALF

After blood collection, mice were sacrificed. The lungs were perfused with PBS using a 20-gauge endotracheal catheter, followed by the collection of the bronchoalveolar lavage fluid (BALF) from lungs. BALF samples were centrifuged at 500 g for 5 min, and the supernatants were stored at -80 °C for further analysis. The pellets were seeded in plate with medium for 3 h and the adherent cells were shown to be alveolar macrophages. The purified alveolar macrophages were used for further analyses.

### Western blot assay

The protein samples from cell lysates were separated using SDS-PAGE and transferred to polyvinylidene fluoride membranes (Bio-Rad, #1,620,177, CA, USA). The membranes were blocked using 5% bovine serum albumin (BSA) for 1 h and incubated with primary antibodies overnight at 4 °C. Those were followed by incubation with corresponding secondary antibodies for 1 h at room temperature. The blots were then visualized using a motored molecular imaging system (Tanon, Shanghai, China).

### RNA extraction and quantitative RT-PCR

Total RNA was extracted from cell lysates according to the EZB RNA reagent kit protocol, and RNA concentration and purity were measured. After total RNA was reversely transcribed to cDNA using HiScript III RT SuperMix (Vazyme, Nanjing, China). PCR was conducted using Taq Pro Universal SYBR qPCR Master Mix (Vazyme, Nanjing, China). Relative mRNA expression was determined using the 2^−ΔCT^ methods relative to the housekeeping gene GAPDH. Data are presented as fold changes of mRNA levels relative to the control groups. The sequences of primers were: IL-6 (5’-ACTTCCATCCAGTTGCCTTCTTGG-3’, 5’-TTAAGCCTCCGACTTGTGAAGTGG-3’), TNFα (5’-GCGACGTGGAACTGGCAGAAG-3’, 5’-GCCACAAGCAGGAATGAGAAGAGG-3’), ATF4 (5’-TCTGCCTTCTCCAGGTGGTTCC-3’, 5’-GCTGCTGTCTTGTTTTGCTCCATC-3’), GDF15 (5’-ATACTCAGTCCAGAGGTGAGAT-3’, 5’-CTTCAGGGGCCTAGTGATG-3’).

### Enzyme-linked immunosorbent assay (ELISA)

The concentrations of IL-6 and TNFα in mouse plasma, BALF, and cell supernatants were measured using ELISA kits (MultiSciences Biotechnology, Hangzhou, China) following the manufacturer’s instructions. The concentrations of GDF15 in mouse plasma, BALF, and cell supernatants were measured using the ELISA kit (#MGD150, R&D Systems, MA, USA). Levels of lactate in the supernatants were measured using L-lactate assay kit (Cat#1,200,011,002, Eton Bioscience, CA, USA).

### Extracellular acidification rate (ECAR) measurement

ECAR was determined using a XF-96 Extracellular Flux Analyzer (Seahorse Bioscience). 8 × 10^4^ MH-S were plated and incubated overnight in Seahorse XF96 Cell Culture Microplates. 1 h prior to the assay media were changed to bicarbonate-free RPMI supplemented with 2mM glutamine and the plate was kept in a non-carbonated incubator. Measurements were performed under basal conditions and after the sequential addition of final 0.5 µM rotenone & antimycin A and 5mM 2DG.

### Statistical analysis

All experiments were independently repeated more than three times. Data were presented as the mean ± SD. All statistical analyses were performed using IBM SPSS Statistical software (26.0, USA), and the experimental results were plotted using Graph Pad Prism software (9.4.1, USA). Any differences between groups were determined using either a two-tailed independent Student’s t-test or one-way analysis of variance (ANOVA) followed with Bonferroni multiple comparison test. *P* < 0.05 was considered to be statistically significant.

## Results

### GDF15 ameliorated sepsis-induced lung injury and inflammation

**Fig. 1:**
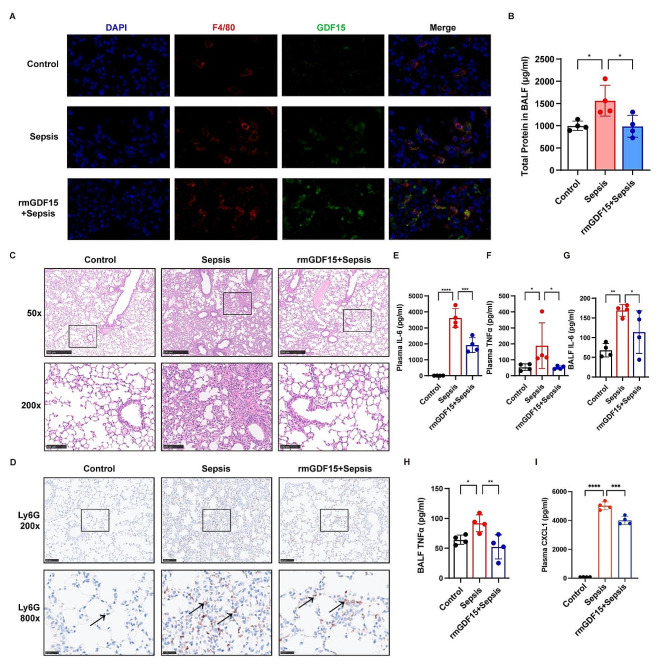
GDF15 ameliorated sepsis-induced lung injury and inflammation. The C57BL/6 mice were intraperitoneally injected with LPS (5 mg/kg) 1 h after rmGDF15 administration (50 ng/kg, i.p.) (*n* = 4). (**A**) Immunofluorescent staining of the co-localization of F4/80 and GDF15 in lung tissues. Scale bar = 10 μm. (**B**) The total protein concentration in BALF was measured. (**C**) H&E staining of lung tissues. (**D**) IHC staining showed the infiltration of Ly6G + neutrophils in lung tissues. (**E-H**) The levels of IL-6 and TNFα in BALF and plasma were tested by ELISA (*n* = 4). (**I**) The levels of CXCL1 in plasma were measured by ELISA (*n* = 4). Data were expressed as the mean ± SD. *p < 0.05, **p < 0.01, ***p < 0.001 and ****p < 0.0001

To investigate the effects of GDF15 on sepsis-induced lung injury in vivo, mice were intraperitoneally injected with recombinant mouse GDF15 (rmGDF15) prior to LPS challenge. Immunofluorescent staining showed that the levels of GDF15 positive macrophages increased after rmGDF15 treatment (Fig. 1A). LPS-induced increase of total protein concentration in the BALF was remarkably inhibited by rmGDF15 (Fig. 1B). Histopathological examination also showed that the lung tissue injury in septic mice was mitigated upon rmGDF15 administration (Fig. 1C). Moreover, rmGDF15 diminished the levels of neutrophil infiltration in septic lung tissues induced by LPS (Fig. 1D). Additionally, the levels of inflammatory cytokines IL-6 and TNFα both in the plasma and the BALF were significantly decreased by rmGDF15 treatment (Fig. 1E-H). Additionally, rmGDF15 decreased the plasma levels of chemokine CXCL1 in septic mice (Fig. 1I). These data suggested that GDF15 alleviates lung injury and inflammation during sepsis.

### AMPK activation-related inhibition of glycolysis and p38/NF-κB signaling mediated the anti-inflammatory effect of GDF15 in AMs

**Fig. 2:**
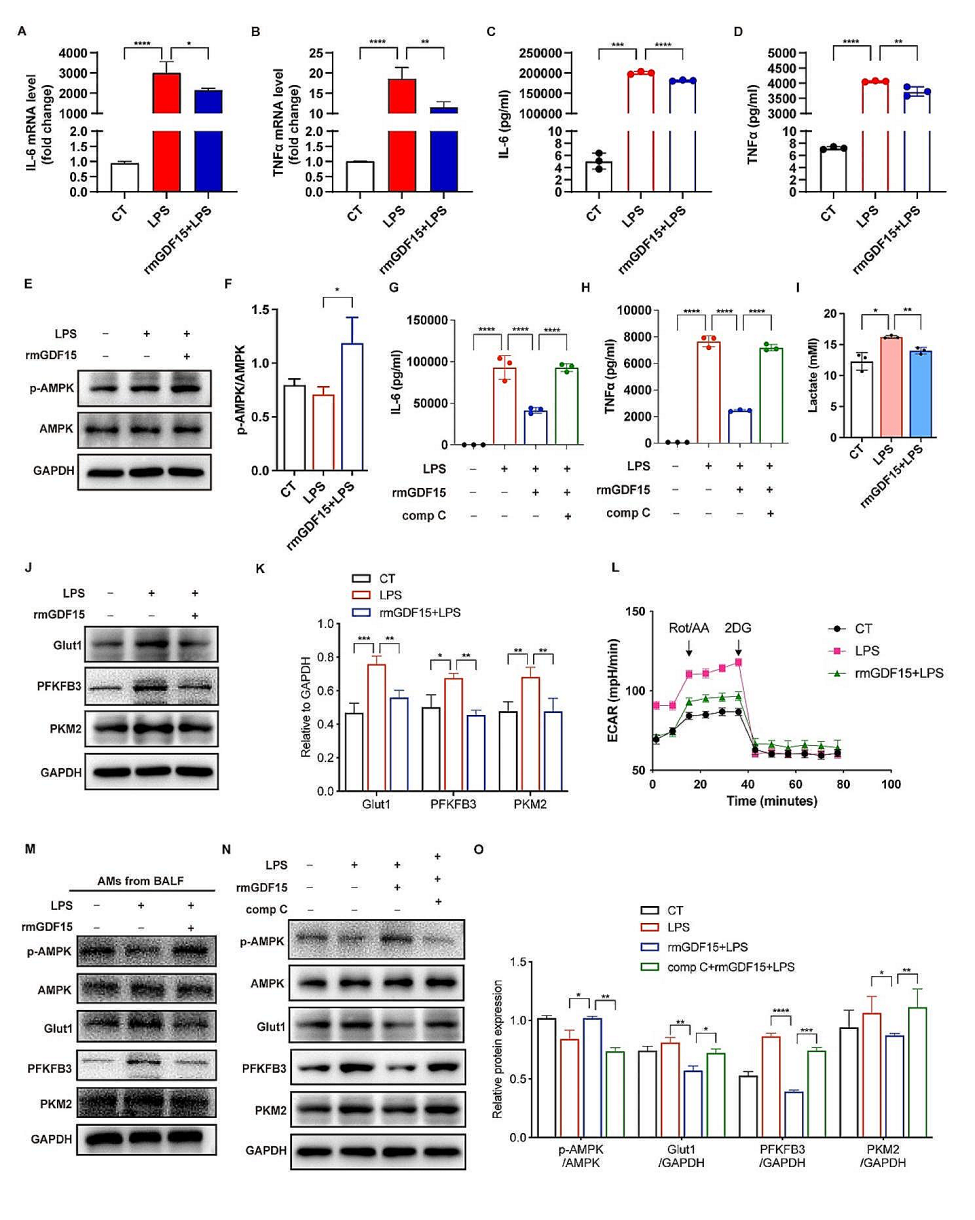
GDF15 inhibited glycolysis via activating AMPK in AMs. (**A, B**) MH-S were pretreated with 2-DG (5 mM) before stimulated with LPS (1 µg/mL) for 6 h. The mRNA expressions of IL-6 and TNFα were determined by RT-qPCR (*n* = 3). (**C, D**) The concentrations of IL-6 and TNFα in the supernatants were measured by ELISA (*n* = 3). (**E, F**) MH-S were pretreated with rmGDF15 (200 ng/mL) prior to LPS (1 µg/mL) stimuli. The protein levels of p-AMPK and AMPK were detected using western blot. (**G, H**) MH-S were pretreated with compound C before rmGDF15 treatment and LPS stimulation. The concentration of IL-6 and TNFα in the supernatants were measured by ELISA (*n* = 3). (**I**) MH-S were pretreated with rmGDF15 prior to LPS stimuli. Lactate concentration in plasma samples was measured (*n* = 3). (**J, K**) The protein levels of glycolytic enzymes were detected using western blot. (**L**) MH-S were pretreated with rmGDF15 before LPS stimuli, followed by the measurement of glycolysis rate for 80 min. Rot&AA: Rotenone & antimycin A; Oligo: Oligomycin. (**M**) The protein levels of phosphorylated AMPK as well as glycolytic enzymes in primary AMs isolated from BALF of septic mice with or without rmGDF15 administration were determined by western blot. (**N-O**) MH-S were pretreated with AMPK inhibitor (compound C, 5 µM) before rmGDF15 treatment and LPS stimulation. The protein levels of phosphorylated AMPK as well as glycolytic enzymes were detected using western blot. comp C: compound C. Data were expressed as the mean ± SD. *p < 0.05, **p < 0.01, ***p < 0.001 and ****p < 0.0001

To verify the role of GDF15 in the inflammatory response of AMs, the alveolar macrophage cell line MH-S were preincubated with rmGDF15 before LPS stimuli. The results showed that LPS-induced upregulations of IL-6 and TNFα were significantly alleviated by rmGDF15 pretreatment (Fig. 2A-D). These data revealed that GDF15 exhibits anti-inflammatory effect on AMs in sepsis.

**Fig. 3:**
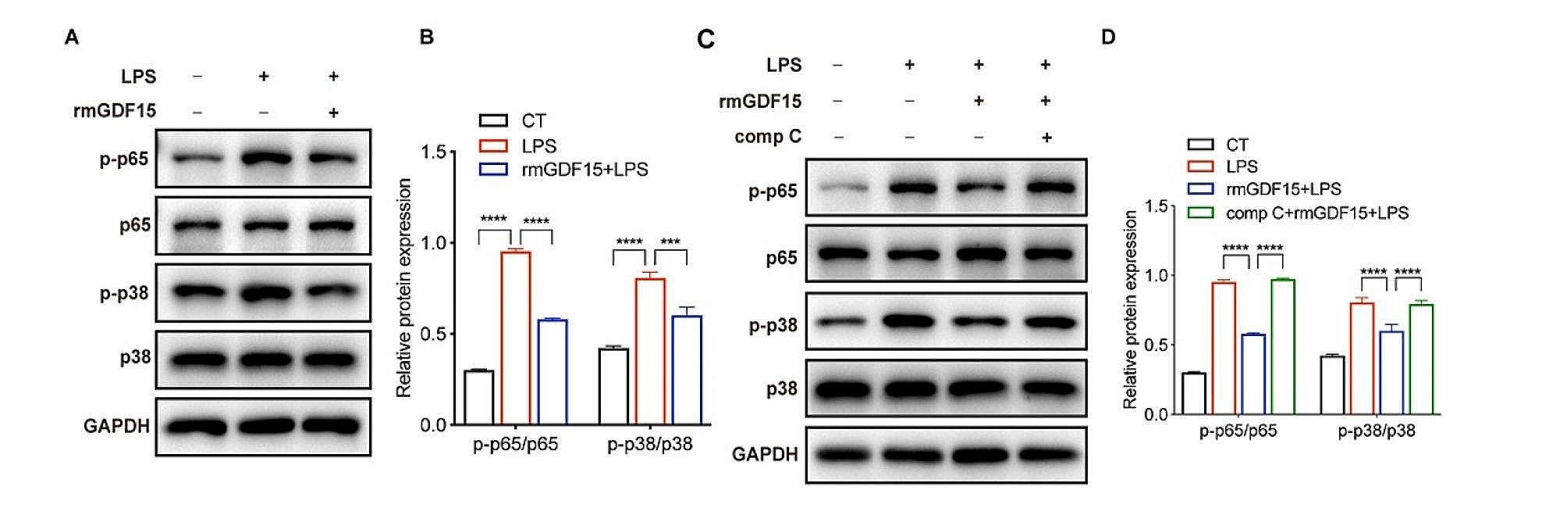
GDF15 inhibited MAPK/NF-κB signaling via AMPK activation. (**A, B**) MH-S were pretreated with rmGDF15 prior to LPS stimuli. The protein levels of p-p65, p65, p-p38, and p38 were detected using western blot. (**C, D**) MH-S were pretreated with compound C before rmGDF15 treatment and LPS stimulation. The protein levels of p-p65, p65, p-p38, and p38 were detected using western blot. Data were expressed as the mean ± SD. *p < 0.05, **p < 0.01, ***p < 0.001 and ****p < 0.0001

Metabolic reprogramming plays a key role in controlling the inflammatory response in macrophages in sepsis [12]. It has been reported that GDF15 as a stress response cytokine regulates energy metabolism via activating AMPK in skeletal muscle [19]. We found that rmGDF15 pretreatment increased the phosphorylation of AMPK in MH-S (Fig. 2E, F). To confirm that GDF15-related anti-inflammatory effect on AMs is mediated by AMPK, MH-S was pre-incubated with compound C, an inhibitor of AMPK, prior to rmGDF15 treatment and LPS stimulation. The results showed that AMPK inhibition diminished rmGDF15-related downregulation of IL-6 and TNFα (Fig. 2G, H). Interestingly, we found that the levels of lactate produced by MH-S upon LPS stimuli were significantly decreased by rmGDF15 treatment (Fig. 2I), suggesting the inhibitory effect of GDF15 on glycolytic metabolism. Accordingly, compared with MH-S treated with LPS, the protein levels of glycolytic enzymes including Glut1, PFKFB3, and PKM2 were decreased by rmGDF15 (Fig. 2J-K). Additionally, rmGDF15 reduced the level of glycolysis in MH-S upon LPS stimuli as indicated by lower extracellular acidification rate (Fig. 2L). Furthermore, the activation of AMPK as well as the inhibition of glycolytic enzymes by rmGDF15 were confirmed in the primary AMs isolated from the BALF of septic mice (Fig. 2M). The inhibition of these glycolytic enzymes by rmGDF15 was reversed by AMPK inhibitor compound C (Fig. 2N-O). Furthermore, nuclear factor kappa-B (NF-κB) and mitogen-activated protein kinases (MAPKs) are key signaling pathways contributing to the inflammatory activation of macrophages. Thus, we examined the effect of GDF15 on the activation of NF-κB and MAPKs. The results showed that rmGDF15 reduced the phosphorylated levels of p65 and p38 (Fig. 3A, B), which was diminished upon AMPK inhibition (Fig. 3C, D). These data indicated that AMPK activation-related inhibition of NF-κB/p38 signaling were involved in GDF15-mediated anti-inflammatory effect on AMs under septic conditions.

### Glycolysis inhibition exhibited anti-inflammatory effect on AMs during sepsis via inducing GDF15 expression

**Fig. 4:**
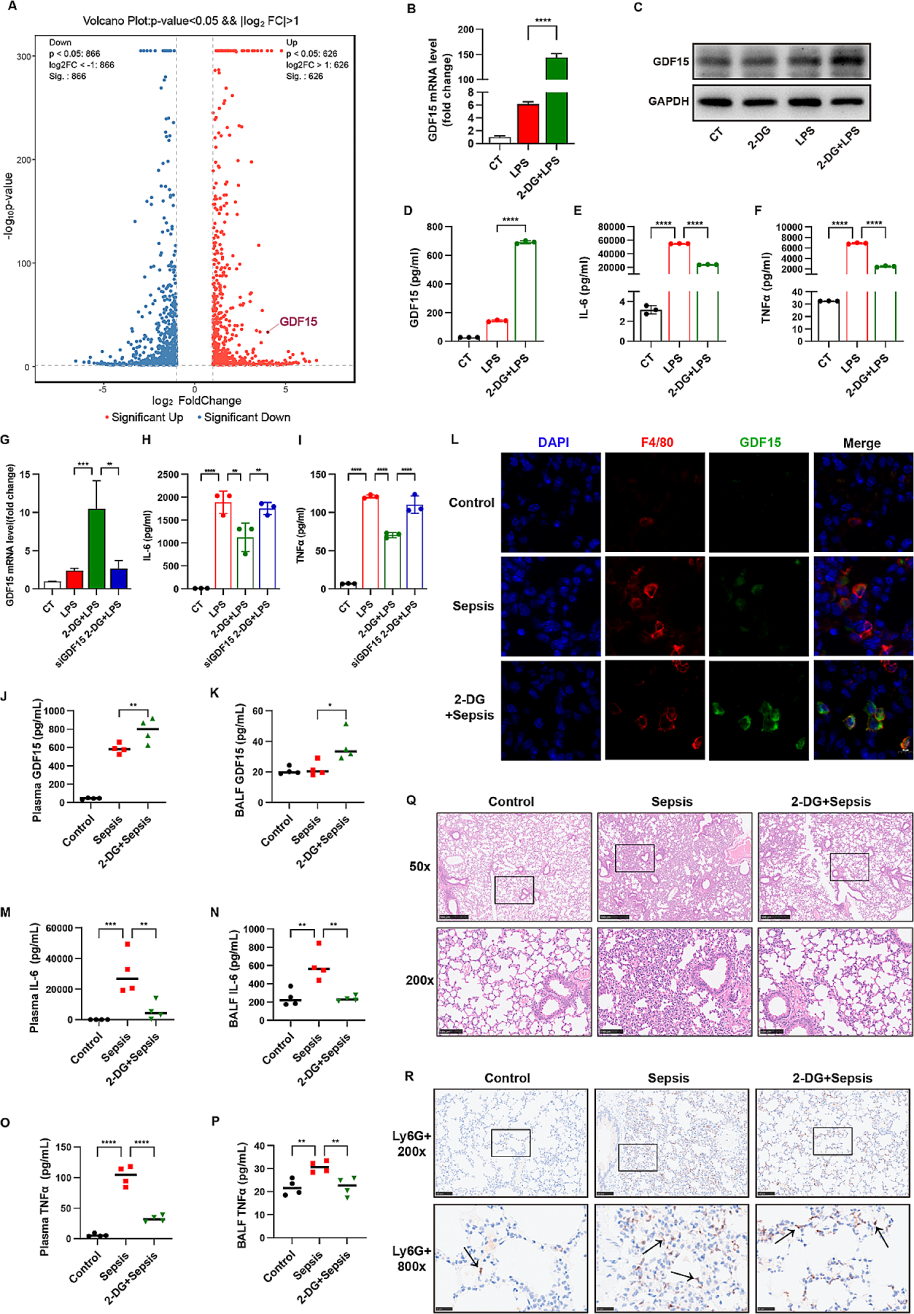
Glycolysis inhibition exhibited anti-inflammatory effect on AMs during sepsis via inducing GDF15 expression. The transcriptome analysis was performed on MH-S stimulated with LPS (1 µg/mL) after 2-DG (5 mM) pretreatment (*n* = 3). (**A**) Volcano plot of differently expressed genes. (**B**) MH-S were stimulated with LPS (1 µg/mL) after 2-DG (5 mM) treatment. The mRNA expressions of GDF15 were detected by RT-qPCR (*n* = 3). (**C**) The protein levels of GDF15 were detected using western blot. (**D**) The secreted levels of GDF15 in the supernatants were measured by ELISA (*n* = 3). (**E, F**) The concentration of IL-6 and TNFα in the supernatants were measured by ELISA (*n* = 3). (**G**) MH-S were transfected with GDF15-specific siRNA or scrambled siRNA before 2-DG (5 mM) pretreatment and LPS (1 µg/mL) stimuli. The mRNA levels of GDF15 were analyzed by RT-qPCR (*n* = 3). (**H, I**) The concentrations of IL-6 and TNFα in the culture medium were measured by ELISA (*n* = 3). (**K, L**) The C57BL/6 mice were i.p. injected with LPS (5 mg/kg) with or without 2-DG (500 mg/kg, i.p.) pretreatment for 1 h. (**J**) Immunofluorescent co-staining of F4/80 and GDF15 in lung tissues. Scale bar = 10 μm. The levels of GDF15 in plasma and BALF were measured by ELISA (*n* = 4). (**M-P**) The levels of IL-6 and TNFα in plasma and BALF were measured by ELISA (*n* = 4). (**Q**) H&E staining of lung tissues. (**R**) IHC staining showed the infiltration of Ly6G + neutrophils in lung tissues. Data were expressed as the mean ± *SD*. *p < 0.05, **p < 0.01, ***p < 0.001 and ****p < 0.0001

Since the plasma levels of GDF15 in patients with sepsis are correlated with the levels of lactate, the product of glycolysis, we speculated the regulatory interaction between GDF15 and glycolytic metabolism. The transcriptome analysis showed that upon the treatment of glycolysis inhibitor 2-DG, the expression of GDF15 in MH-S was significantly upregulated (Fig. 4A). To further examine the response of GDF15 to glycolysis inhibition, MH-S was treated with 2-DG prior to LPS stimulation. The results showed that 2-DG pretreatment significantly increased the expression of GDF15 at the mRNA levels as well as the intracellular and extracellular protein levels (Fig. 4B-D). In addition, 2-DG treatment dramatically reduced the levels of cytokines IL-6 and TNF⍺ produced by AMs (Fig. 4E, F). To examine whether GDF15 mediates the anti-inflammatory effect of 2-DG on AMs, GDF15 was knocked down in MH-S before 2-DG treatment and LPS stimulation. The results showed that the downregulations of IL-6 and TNF⍺ by 2-DG were reversed in GDF15-deficient MH-S (Fig. 4G-I). In the septic mouse model in vivo, 2-DG administration effectively increased the levels of GDF15 both in the plasma and BALF (Fig. 4J, K). Immunofluorescent staining showed that the levels of GDF15 were increased in macrophages in the lung tissues of septic mice treated with 2-DG compared to that in septic mice (Fig. 4L). Simultaneously, LPS challenge-induced inflammatory responses both in the plasma and BALF and lung tissue injury were alleviated by 2-DG treatment (Fig. 4M-P). Additionally, the lung injury as well as the infiltration of neutrophils in septic lung tissues was diminished in septic mice treated with 2-DG (Fig. 4R). These data revealed that GDF15 mediates the anti-inflammatory effect of 2-DG on AMs during sepsis.

### eIF2⍺-ATF4 signaling mediated 2-DG-related GDF15 expression in AMs

**Fig. 5:**
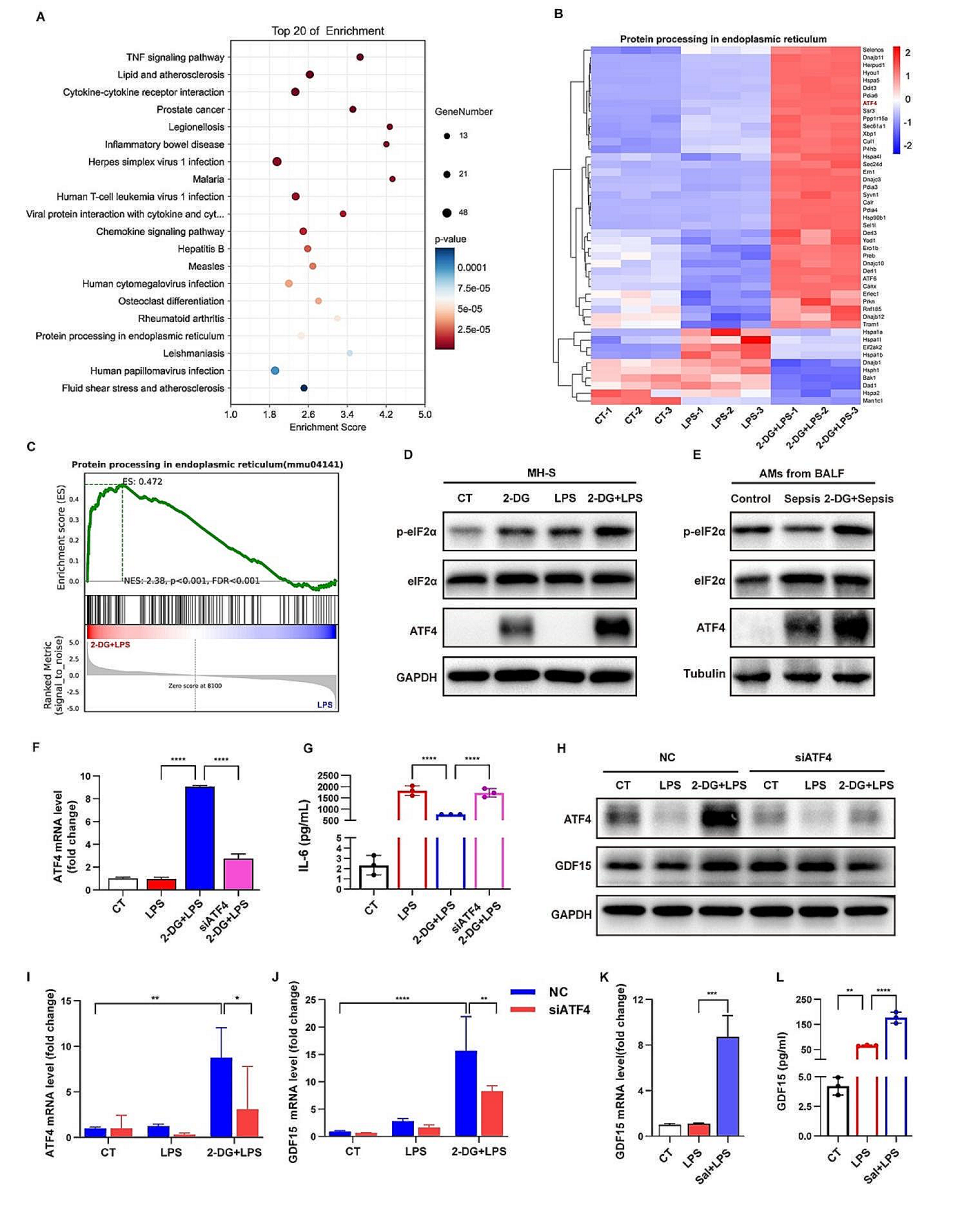
eIF2⍺-ATF4 signaling mediated 2-DG-related GDF15 expression in AMs. (**A**) KEGG analysis of genes regulated by both LPS stimulation and 2-DG treatment. (**B**) Heatmap of differently expressed genes in the pathway related to protein processing in endoplasmic reticulum (ER). (**C**) GSEA analysis showing the primary enrichment of ER stress signaling in 2-DG-regulated genes. (**D**) The protein levels of phosphorylated eIF2α and ATF4 in LPS-stimulated MH-S with or without 2-DG pretreatment were analyzed by western blot. (**E**) The protein levels of phosphorylated eIF2α and ATF4 in AMs isolated from BALF of septic mice with or without 2-DG pretreatment were analyzed by western blot. (**F, G**) MH-S were transfected with ATF4-specific siRNA or scrambled siRNA before 2-DG (5 mM) pretreatment and LPS (1 µg/mL) stimuli. The mRNA level of ATF4 was detected by RT-qPCR (*n* = 3). The concentrations of IL-6 in the culture medium were measured by ELISA (*n* = 3). (**H-J**) The mRNA levels of ATF4 and GDF15 were detected by RT-qPCR (*n* = 3). The protein levels of GDF15 in MH-S were detected by western blot. (**K, L**) MH-S were treated with LPS (1 µg/ml) after Salubrinal (50 µM) pretreatment. The mRNA level of GDF15 were detected by RT-qPCR (*n* = 3). The secreted levels of GDF15 in the supernatants were measured by ELISA (*n* = 3). Sal = Salubrinal. Data were expressed as the mean ± *SD*. *p < 0.05, **p < 0.01, ***p < 0.001 and ****p < 0.0001

The Kyoto-Encyclopedia of Genes and Genomes (KEGG) analysis of the transcriptome data showed that 2-DG-regulated genes were significantly enriched in the signaling pathway associated with the protein processing in endoplasmic reticulum (ER) (Fig. 5A). The heatmap showed the expressions of genes enriched in protein processing in endoplasmic reticulum (ER) were significantly converted by 2-DG treatment under the septic condition (Fig. 5B). Also, the gene set enrichment analysis (GSEA) showed that the highly expressed genes in 2-DG-treated group were enriched in the signaling pathway related to protein processing in ER (Fig. 5C). These data suggests that ER stress activation may be involved in 2-DG-related regulation of AMs under septic conditions.

The eukaryotic translation initiation factor-2α (eIF2α)-ATF4 signaling plays an important role in the activation of ER stress. To verify the effect of 2-DG on eIF2α-ATF4 signaling in AMs, MH-S were treated with 2-DG before LPS stimulation. The results showed that the levels of phosphorylated eIF2α as well as the expression of ATF4 were dramatically induced by pretreatment of 2-DG (Fig. 5D). To verify the effect of 2-DG on eIF2α-ATF4 signaling in vivo, AMs were isolated from the BALF of septic mice challenged with LPS in the absence or presence of 2-DG treatment. The results showed that 2-DG effectively increased the levels of phosphorylated eIF2α and ATF4 in AMs isolated from BALF (Fig. 5E). To further investigate whether the anti-inflammatory effect of 2-DG is mediated by ER stress activation, ATF4 was knocked down in MH-S before 2-DG treatment and LPS stimuli. The downregulation of IL-6 by 2-DG was effectively reversed upon the deletion of ATF4 (Fig. 5F, G). These data indicated that ATF4 plays an important role in mediating the anti-inflammatory effect of 2-DG in AMs under septic conditions.

To examine the mediatory role of eIF2⍺-ATF4 signaling in 2-DG-related induction of GDF15, ATF4 was knocked down in MH-S before 2-DG treatment and LPS stimuli. The results showed that ATF4 deficiency significantly abolished the upregulation of GDF15 by 2-DG (Fig. 5H-J). Additionally, the dephosphorylation inhibitor of eIF2α, salubrinal, remarkably increased the expression of GDF15 in MH-S, confirming the upregulation of GDF15 by eIF2⍺-ATF4 signaling activation (Fig. 5K, L). These data revealed that 2-DG induces GDF15 expression in AMs by activating eIF2⍺-ATF4 signaling.

### GDF15 is closely correlated with the severity of septic patients

**Fig. 6:**
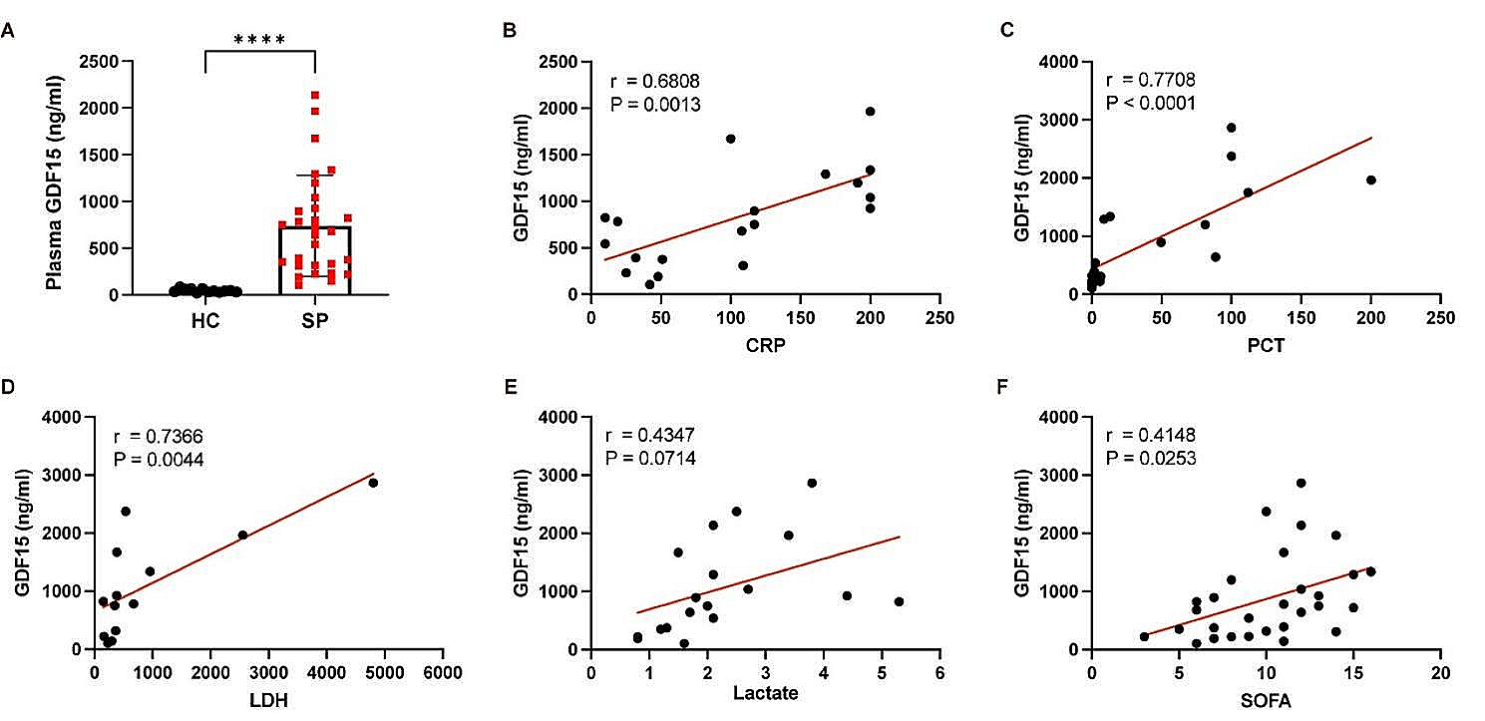
The increased plasma levels of GDF15 in patients with sepsis were positively correlated with clinical prognostic indicators. (**A**) The plasma levels of GDF15 in septic patients and healthy controls were measured by ELISA. (**B-F**) Correlation analysis of plasma GDF15 and CRP, PCT, LDHA, lactate, and SOFA scores in patients with sepsis

We further examined the plasma levels of GDF15 in septic patients and healthy controls. The clinical data of all subjects are shown in Table 1. We observed that the concentrations of GDF15 in septic patients were significantly higher compared to healthy controls (Fig. 6A). Furthermore, the plasma levels of GDF15 in septic patients were positively correlated with the plasma levels of CRP (*P* = 0.0013) and PCT (*P* < 0.0001) (Fig. 6B, C). The levels of LDH and lactate in the plasma also showed positive correlations with the plasma levels of GDF15 (*P* = 0.0044 and 0.0714) (Fig. 6D, E). Additionally, there was a significantly correlation between GDF15 levels and SOFA scores with the coefficient 0.4148 (*P* = 0.0253) (Fig. 6F). The above data indicated that GDF15 is closely associated with the severity of patients with sepsis.

**Table 1:** Characteristics of septic patients and healthy controls

	Healthy controls (*n* = 12)	Septic patients (*n* = 29)
Gender(male/female)	7/5	20/9
Age (years)	59.17 ± 13.84	61.59 ± 15.756
WBC (×10^9^/L)	-	14.62(7.30, 20.17)
PLT	-	190.50(86.00, 298.75)
Hb	-	110.00(83.75, 161.75)
D-Dimmer	-	0.73(0.35, 3.74)
PA	-	62.00(51.25, 158.25)
PCT	-	0.22(0.08, 15.21)
CRP	-	112.00(49.50, 200.00)
LDH	-	333.50(250.25, 2007.50)
Lactate	-	2.00(1.68, 3.08)
IL-6	-	156.80(10.70, 476.90)
SOFA	-	10.50(7.00, 13.25)
APACHEII	-	17.50(17.00, 18.75)

WBC: white blood cell; PLT: Platelet count; Hb: Hemoglobin; PA: Prealbumin; PCT: procalcitonin; CRP: C-reactive protein; LDH: lactate dehydrogenase; SOFA: sequential organ failure assessment; APACHE: acute physiology and chronic health evaluation

## Discussion

**Fig. 7:**
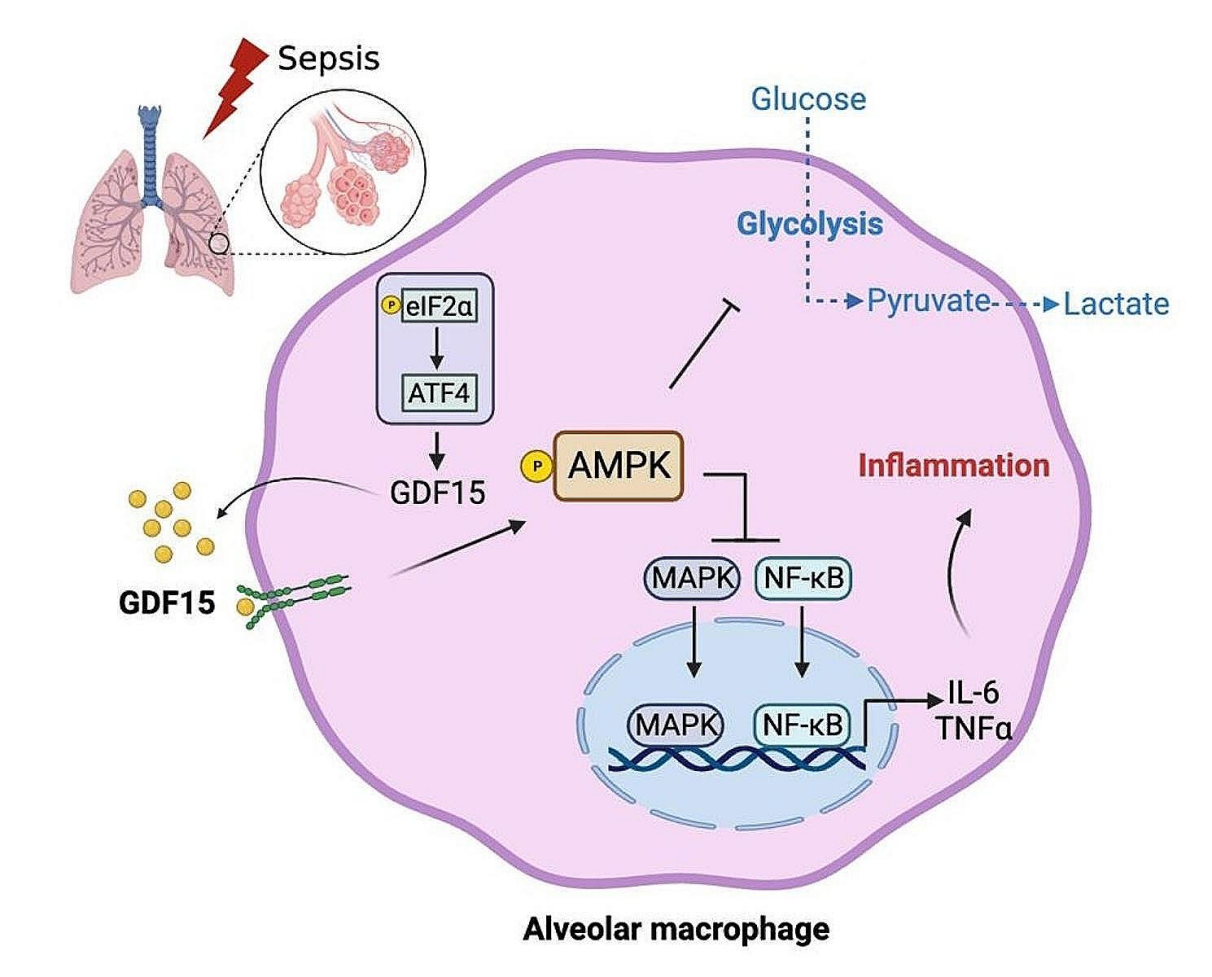
Schematic illustration of GDF15 regulation in sepsis-induced inflammatory response of AMs. Glycolysis inhibition promoted GDF15 expression via activating eIF2⍺-ATF4 axis. In turn, GDF15 inhibited glycolysis and MAPKs/NF-κB signaling through AMPK activation, thereby alleviating the inflammatory response and lung injury in sepsis

Sepsis-induced lung injury is a severe inflammatory disorder characterized by a dramatic cytokine storm as well as the excessive lung infiltration of macrophages and neutrophils into the lung tissue [20]. AMs within alveoli serving as immune surveillance is the first line of protection against lung infection [21]. Overwhelming activation of AMs leads to the production of pro-inflammatory cytokines, followed by alveolar epithelial disruption and lung injury. Previous studies have demonstrated the association of GDF15 with the prognosis of septic patients as well as its controversial roles in sepsis-related tissue damage [22, 23]. In the current study, we provided a better understanding of GDF15 in sepsis-induced lung injury. We found that plasma levels of GDF15 were positively correlated with the severity of septic patients. Supplementation of GDF15 diminished inflammation and lung injury in LPS-induced mouse sepsis model. Glycolysis inhibition by 2-DG promoted GDF15 expression and secretion via activating eIF2α-ATF4 signaling. In turn, GDF15, by increasing AMPK phosphorylation, inhibited glycolysis and NF-κB/MAPKs, thereby abolishing the inflammatory response of AMs (Fig. 7). Our findings imply that GDF15 might serve as a potential target for sepsis-related lung injury therapies.

Glycolysis and ER stress have been considered to modulate the biological functions of macrophages in several diseases. However, the views with regard to the interconnection between glycolysis and ER stress are controversial. ER stress and the unfolded protein response (UPR) activation has been reported to upregulate glycolytic enzymes and downregulate genes encoding TCA cycle and mitochondrial respiratory chain enzymes in Drosophila melanogaster S2 cells [24]. Glycolytic intermediates promote protein N-glycosylation for enabling protein folding and processing in the ER [25, 26]. Additionally, LDHA inhibition by oxamate showed anti-fibrotic role by reducing glycolysis and ER stress in silicotic mice [27]. On the contrary, it has been reported that glycolysis-derived pyruvate ameliorated high glucose-induced ER stress in HK-2 cells via downregulating the expression of CHOP, ATF4, and phosphorylated eIF2⍺ [28]. In addition, IRE1-mediated UPR signaling activation reduced glucose metabolism as part of an adaptive response [29]. In our study, we found that glycolysis inhibitor 2-DG activated the ER stress by inducing eIF2⍺-ATF4 signaling, thereby promoting GDF15 expression to exhibit anti-inflammatory effect on AMs. These contradictive findings might be caused by different cell types or disease models. It is worthy to further explore the interaction between glycolysis and ER stress in macrophages under septic conditions.

GDF15 expression increases in many pathological conditions and serves as a marker of cellular stress and injury [11, 30]. GDF15 is a biomarker with prognostic importance in patients with sepsis and COVID-19. The increased plasma levels of GDF15 were positively correlated with the severity and endpoint of intensive care unit admission or death [31]. GDF15 has paradoxical roles within a pathological condition. Our data showed that GDF15 effectively limited AM inflammation and lung injury by inhibiting glycolysis and classical inflammatory signaling in sepsis. Herein, we also observed significantly increased levels of GDF15 in septic mice and septic patients. In contrast to the beneficial effect of GDF15, it has been reported that GDF15 deficiency limits neutrophil recruitment to the site of infection and delays local pathogen control and clearance in bacterial infection [23]. The contradictive effects of GDF15 can be dose and time related and also depends on the targeted tissues. It should be noted that pro-inflammatory and anti-inflammatory macrophages exist simultaneously in the septic lung tissues. Thus, further evaluation of macrophage functions after rmGDF15 treatment are required to better understand the beneficial effect of GDF15 on macrophage modulation.

GDF15 is widely investigated in several metabolic dysfunctions, including obesity, diabetes, and fatty liver diseases [32]. It has been observed that the metabolic effects of pharmacological activation of peroxisome proliferator-activated receptor β/𝛿 (PPARβ/𝛿) on glucose intolerance and fatty acid oxidation were mediated by GDF15 expression [19]. In nonalcoholic fatty liver disease (NAFLD), GDF15 levels are elevated in proportion to the severity of the disease [33]. GDF15 gene deletion increases lipid accumulation in the mouse liver. In sepsis, metabolic reprogramming favoring aerobic glycolysis contributes to the pro-inflammatory phenotype of macrophages. We discovered that GDF15 limited the glycolytic flux in AMs and mediated the beneficial effects of 2-DG on inflammatory response of AMs in sepsis-induced lung injury. Our study provided the first evidence that GDF15 interplay with glycolysis to ameliorate sepsis-induced inflammation in AMs. The metabolic characteristic of AMs in the lung during sepsis is controversial. It has been reported that LPS induced the glycolytic metabolism of AMs, which was blunted by saturated fatty acid palmitate [34]. In addition, the M1 polarization and glycolysis of alveolar macrophage was induced by FoxO3-mediated activation of PFKFB3 in sepsis-related acute lung injury [35]. On the other hand, it has been reported that glycolysis is dispensable for macrophage effector function in alveolar macrophages [36]. For interstitial macrophages, glycolysis has been recognized as the main metabolic contribution to the inflammatory activation upon LPS stimuli. However, it has been recently demonstrated that activated bone marrow-derived macrophages showed both increased glycolysis and tricarboxylic acid (TCA) cycle volume. Inhibition of mitochondrial metabolism also alleviated the inflammatory response of macrophages [37]. Therefore, the role of glycolysis in sepsis-induced inflammatory response of AMs needs to be further clarified to better understand the metabolic reprogramming characteristics during sepsis.

In conclusion, our study demonstrated that glycolysis inhibition promoted GDF15 expression in AMs via activating eIF2⍺-ATF4 axis. In turn, GDF15 inhibited glycolysis and NF-κB/MAPKs signaling through AMPK activation, thereby alleviating the inflammatory response and lung injury in sepsis. Our findings provided potentially novel therapeutic strategies for the treatment of sepsis-associated lung injury.

### Electronic supplementary material

Below is the link to the electronic supplementary material.

Supplementary Material 1

## Data Availability

The datasets generated during and/or analyzed during the current study are available from the corresponding author on reasonable request.
