# Supplementary material for: GDF15 ameliorates sepsis-induced lung injury via AMPK-mediated inhibition of glycolysis in alveolar macrophage
Source: Respir Res. 2024 May 9;25:201. doi: 10.1186/s12931-024-02824-z (PMC11084091; doi:10.1186/s12931-024-02824-z)
Supplement: Supplementary file 1 — Supplementary Material 1 [file 12931_2024_2824_MOESM1_ESM.pdf]

Western blot raw data

Figure 2

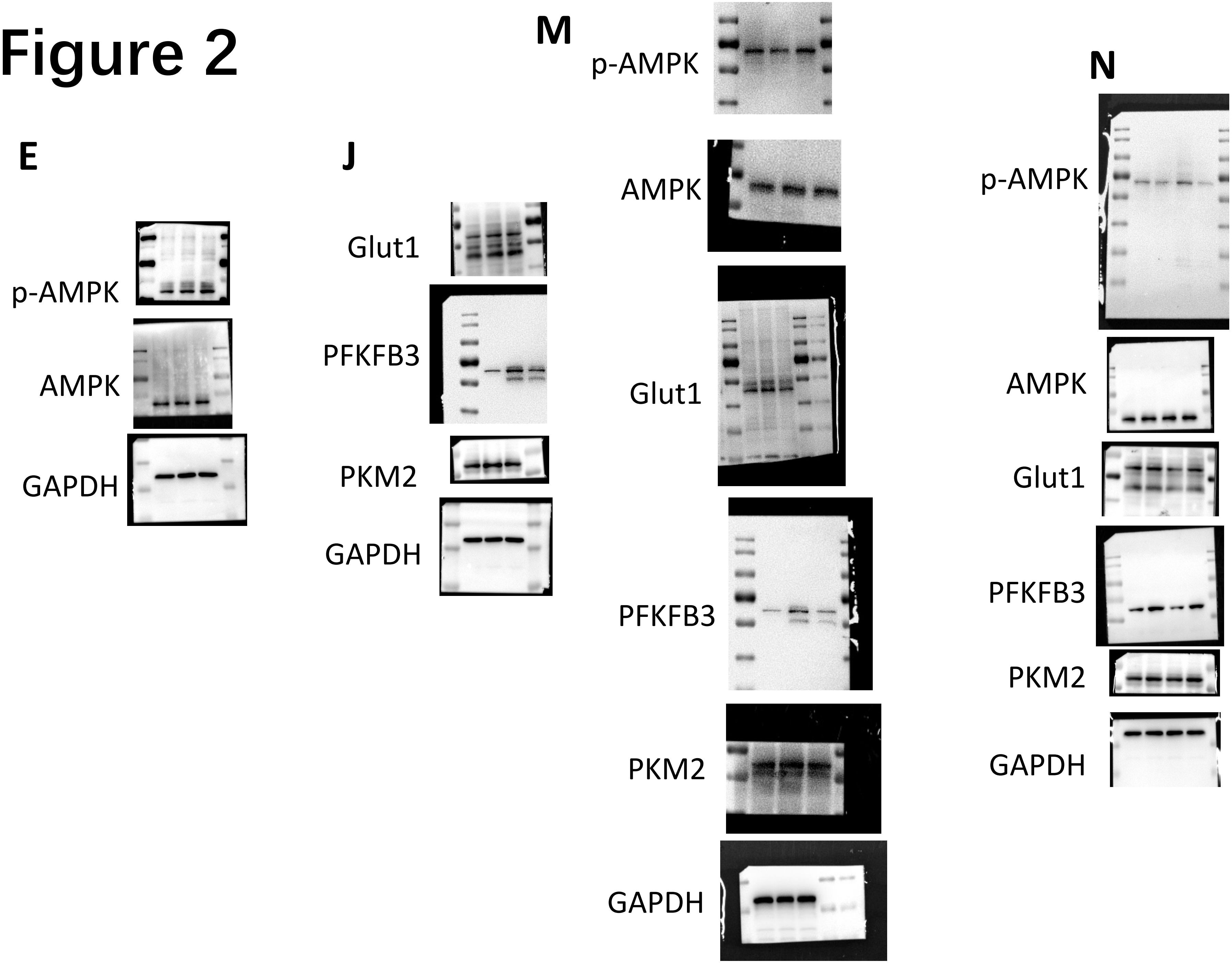

E
p-AMPK
AMPK
GAPDH
J
Glut1
PFKFB3
PKM2
GAPDH
M
p-AMPK
AMPK
Glut1
PFKFB3
PKM2
GAPDH
N
p-AMPK
AMPK
Glut1
PFKFB3
PKM2
GAPDH

Figure 3

A

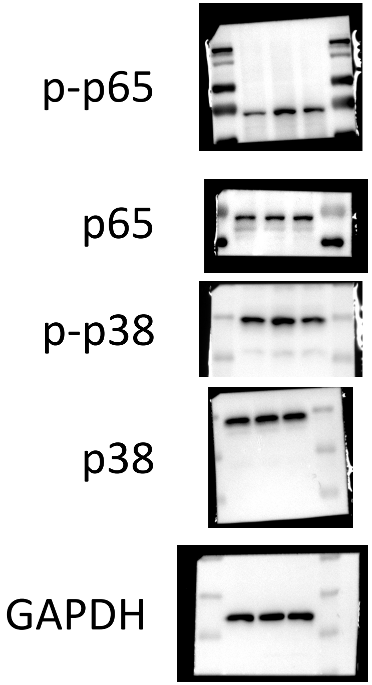

C

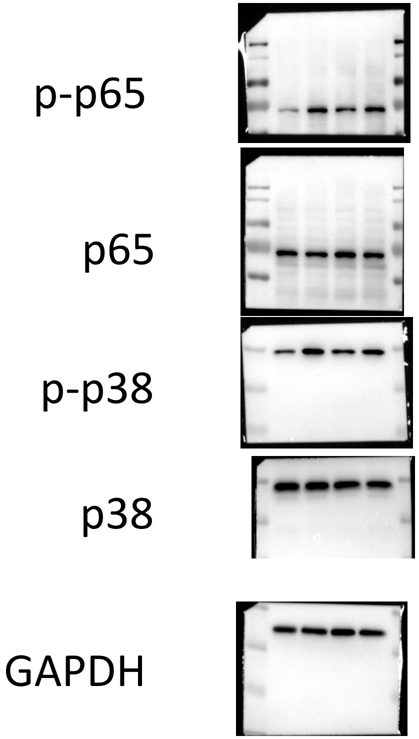

Figure 4

c

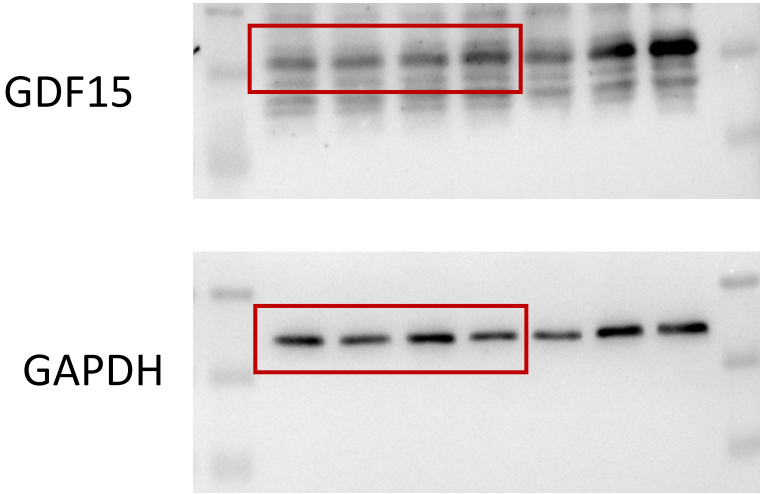

Figure 5

D

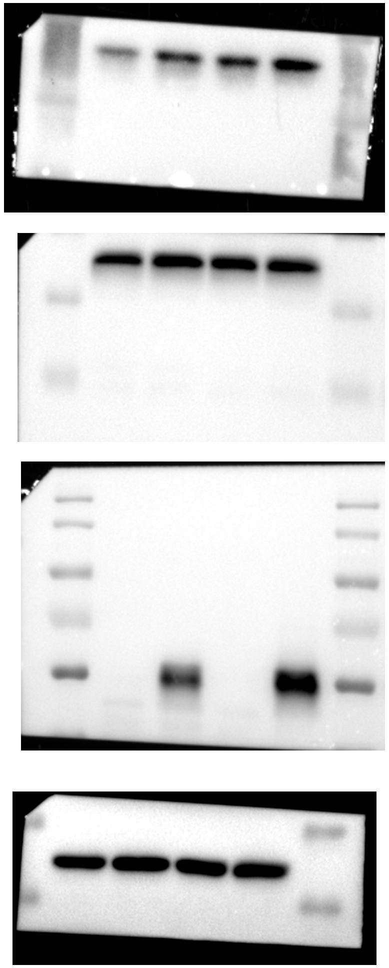

P-eIF2α
eIF2α
ATF4
GAPDH

E

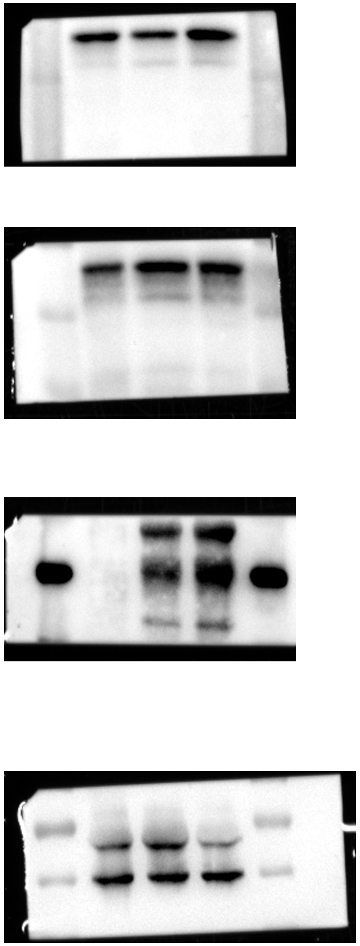

P-eIF2α
eIF2α
ATF4
GAPDH

H

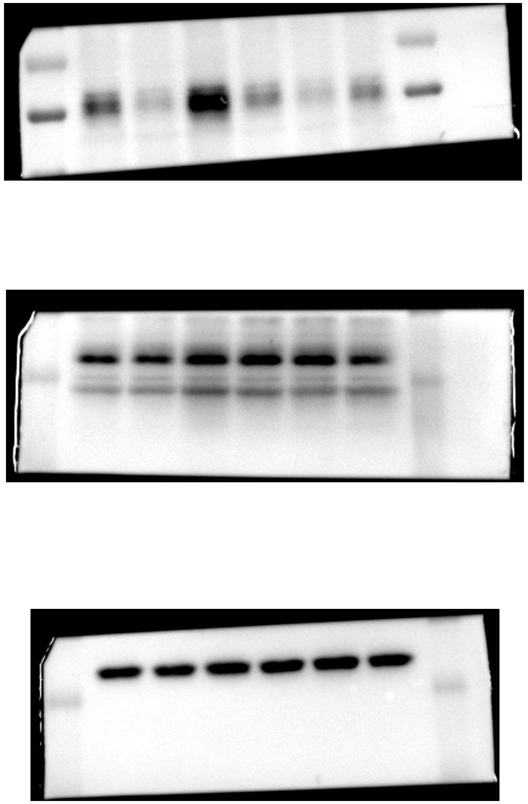

ATF4
GDF15
GAPDH
